# Biodegradable Cellulose/Polycaprolactone/Keratin/Calcium Carbonate Mulch Films Prepared in Imidazolium-Based Ionic Liquid

**DOI:** 10.3390/polym15122729

**Published:** 2023-06-18

**Authors:** Dušica Stojanović, Aleksandra Ivanovska, Nemanja Barać, Katarina Dimić-Misić, Mirjana Kostić, Vesna Radojević, Djordje Janaćković, Petar Uskoković, Ernest Barceló, Patrick Gane

**Affiliations:** 1 Faculty of Technology and Metallurgy, University of Belgrade, Karnegijeva 4, 11000 Belgrade, Serbia; duca@tmf.bg.ac.rs (D.S.); kostic@tmf.bg.ac.rs (M.K.); vesnar@tmf.bg.ac.rs (V.R.); nht@tmf.bg.ac.rs (D.J.); puskokovic@tmf.bg.ac.rs (P.U.); 2 Innovation Center of the Faculty of Technology and Metallurgy in Belgrade Ltd., University of Belgrade, Karnegijeva 4, 11000 Belgrade, Serbia; nbarac@tmf.bg.ac.rs; 3 Department of Bioproducts and Biosystems, School of Chemical Engineering, Aalto University, 00076 Helsinki, Finland; katarina.dimic-misic@metsagroup.com (K.D.-M.); ebarcelorodriguez@gestamp.com (E.B.)

**Keywords:** mulch films, cellulose, polycaprolactone, calcium carbonate, keratin, waste chicken feathers, biodegradation

## Abstract

Ionic liquid 1-butyl-3-methylimidazolium chloride [BMIM][Cl] was used to prepare cellulose (CELL), cellulose/polycaprolactone (CELL/PCL), cellulose/polycaprolactone/keratin (CELL/PCL/KER), and cellulose/polycaprolactone/keratin/ground calcium carbonate (CELL/PCL/KER/GCC) biodegradable mulch films. Attenuated Total Reflectance Fourier-Transform Infrared (ATR-FTIR) spectroscopy, optical microscopy, and Field-Emission Scanning Electron Microscopy (FE-SEM) were used to verify the films’ surface chemistry and morphology. Mulch film made of only cellulose regenerated from ionic liquid solution exhibited the highest tensile strength (75.3 ± 2.1 MPa) and modulus of elasticity of 944.4 ± 2.0 MPa. Among samples containing PCL, CELL/PCL/KER/GCC is characterized by the highest tensile strength (15.8 ± 0.4 MPa) and modulus of elasticity (687.5 ± 16.6 MPa). The film’s breaking strain decreased for all samples containing PCL upon the addition of KER and KER/GCC. The melting temperature of pure PCL is 62.3 °C, whereas that of CELL/PCL film has a slight tendency for melting point depression (61.0 °C), which is a characteristic of partially miscible polymer blends. Furthermore, Differential Scanning Calorimetry (DSC) analysis revealed that the addition of KER or KER/GCC to CELL/PCL films resulted in an increment in melting temperature from 61.0 to 62.6 and 68.9 °C and an improvement in sample crystallinity by 2.2 and 3.0 times, respectively. The light transmittance of all studied samples was greater than 60%. The reported method for mulch film preparation is green and recyclable ([BMIM][Cl] can be recovered), and the inclusion of KER derived by extraction from waste chicken feathers enables conversion to organic biofertilizer. The findings of this study contribute to sustainable agriculture by providing nutrients that enhance the growth rate of plants, and hence food production, while reducing environmental pressure. The addition of GCC furthermore provides a source of Ca^2+^ for plant micronutrition and a supplementary control of soil pH.

## 1. Introduction

The continuous growth of the world population in the 21st century inevitably led to increased food demand and enhanced production. Taking into account the limited availability of arable land, both efficient and sustainable agricultural practices become big challenges [1]. On the other hand, advanced technologies enable the detection of numerous agricultural problems and contribute to the application of new technologies to solve existing problems. The end-of-life management of agricultural plastic, i.e., avoiding its improper disposal, is one of the most worrisome problems, since only 46% of agricultural plastic waste is recycled or recovered [2]. The worldwide use of agricultural plastic films mainly includes films used for greenhouses (grow tunnels), fodder wrapping, e.g., silage, and mulching. Mulching improves crop yield, decreases pesticide input to the soil, saves irrigation water, and so contributes significantly to tackling the increase in food demand [3,4]. Although diverse materials are nowadays used for mulching, the vast majority of plastic mulch is composed of polyethylene, a highly durable, resistant, and cheap plastic based on fossil fuels. However, polyethylene’s long life in the environment results in the contamination of agricultural soils with its residues, which represents a serious environmental concern. Furthermore, the economic cost of the yearly removal and disposal of such mulch residues should not be neglected.

Although mulch films have been studied for decades, the production routes for obtaining mulch films alternative to polyethylene ones, which can be degraded by the action of microorganisms present in the soil, are nowadays much more intensively studied. To achieve this, almost inexhaustible naturally occurring materials, such as cellulose, are employed. Due to the limited solubility of cellulose in many of the chemical agents classically used in industrial production, much focus has been given to obtaining this biodegradable biopolymer from renewable sources using green chemistry. One such method is the recovery of regenerated cellulose by dissolution in ionic liquids, such as 1-butyl-3-methylimidazolium chloride [BMIM][Cl] [5,6]. Imidazolium-based ionic liquid has also been observed to be capable of dissolving some other biopolymers, including, for example, keratin, thus allowing the manufacturing of bi- or multicomponent mulch films with combined functionalities [5,7]. Keratin has recently gained much attention for the preparation of mulch films since it can be extracted from the most abundant locally available keratin source, e.g., chicken feather waste, which is usually either disposed of in landfills, used as low-nutritional-value animal feed, or incinerated [5]. By its conversion into a high-value component in fertilizers, keratin can serve as a nutrient for plants due to its high nitrogen content, proteins, amino acids, sulfur, carbon, and other elements [8], reducing environmental pressure and improving the quality of the soil for sustainable agriculture [9]. The relatively poor mechanical properties of keratin in mulch film formulations can be overcome by adding calcium carbonate (CaCO_3_), a readily available natural functional filler that promotes the crystallization of the polymer by seeding many small nuclei. In parallel, it can impart whiteness that will reduce soil warming due to the resulting albedo effect and itself constitutes a plant micronutrient calcium source as well as providing a soil pH buffer action. Besides the addition of CaCO_3_, the tensile properties of the blends can be adjusted by incorporating various amounts of the most common polyester, partially crystalline polycaprolactone (PCL) [10], into mulch films. The carbonyl groups of this thermoplastic synthetic polymer can form hydrogen bonds with cellulose hydroxyl groups, which can be used to affect the polymer blend morphology and other properties [10]. It has to be noted that PCL is degradable in soil; the hydrolyzable ester linkage of this linear aliphatic polymer makes it susceptible to microbial degradation via lipase and esterase action [11]. Additionally, PCL in combination with keratin forms composites that serve as carriers in controlled and slow-release applications of nutrients, fertilizers, and pheromones [12].

The above considerations prompted us to initiate this study, which aims to prepare mulch films that can be valued for their low cost, relative biodegradability, and processability. To achieve this, as described above, readily available naturally occurring materials such as cellulose, keratin, and calcium carbonate, as well as biodegradable polycaprolactone, were employed. Ionic liquid [BMIM][Cl] was used to prepare cellulose, cellulose/polycaprolactone, cellulose/polycaprolactone/keratin, and cellulose/polycaprolactone/keratin/ground calcium carbonate mulch films. ATR-FTIR spectroscopy, optical microscopy, and FE-SEM were used to verify the surface chemistry and morphology of the resulting films. Thereafter, the films with their various component blends were characterized in terms of their mechanical and optical properties. Simulated degradation in the soil was then applied to verify the environmental feasibility of adopting them as mulch films. The outcomes of this study will be beneficial in the ongoing development of biodegradable, micronutrient-delivering mulch films and their potential for commercialization, especially since, to the authors’ knowledge, the combination of cellulose/polycaprolactone/keratin/ground calcium carbonate has not been reported elsewhere.

## 2. Materials and Methods

### 2.1. Materials

Cellulose (CELL) was obtained by grinding prehydrolyzed Kraft birch pulp sheets (*M*_w_ = 262.9 kDa, polydispersity of 3.6, Stora Enso Enocell, Finland). PCL (*M*_w_ = 80,000 g mol^−1^) was purchased from Sigma-Aldrich, Saint Louis, MO, USA. 1-butyl-3-methylimidazolium chloride [BMIM][Cl] (assay 99%, *M*_w_ = 174.67 g mol^−1^, melting point of 65 °C) was obtained from IoLiTec Ionic Liquids Technologies GmbH (Heilbronn, Germany). Ground CaCO_3_ (GCC) (ISO brightness ≥ 96%) made from Norwegian marble without the use of dispersing agent, and so chemical-free, was supplied by Omya Hustadmarmor AS (Elnesvågen, Norway). The GCC particle size distribution was quoted according to the proportional volume of sample below a given particle diameter determined by time-average light scattering, *d*_%(vol)_, as follows: *d*_10(vol)_ ≤ 3.33 µm; *d*_50(vol)_ ≤ 4.61 µm; *d*_90(vol)_ ≤ 6.05 µm. To remove moisture, before the experiments, CELL and [BMIM][Cl] ionic liquid were dried at 70 °C under vacuum for about 12 h, while ground PCL pellets were dried for the same time at room temperature.

### 2.2. Extraction of Keratin from Chicken Feather Waste

**Figure 1:**
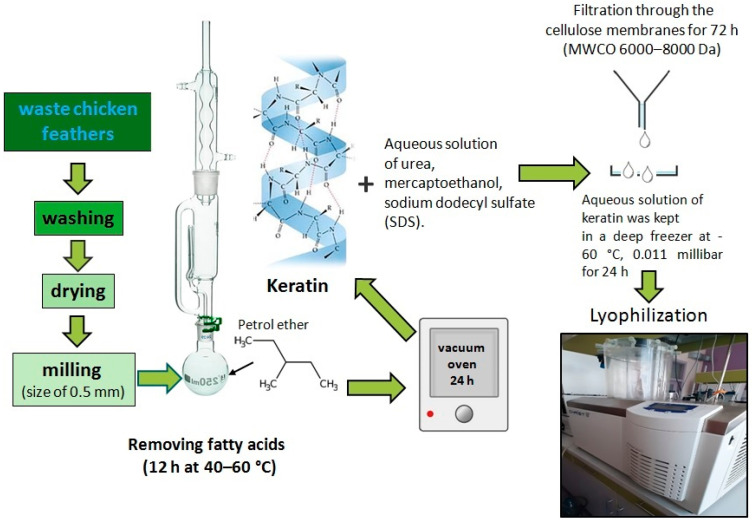
Extraction of keratin from chicken feather waste.

Keratin was obtained from chicken feather waste (derived from Perutnina Ptuj, Slovenia) through the extraction process detailed in a previously published procedure [13], Figure 1. The keratin aqueous solution was freeze-dried at −60 °C at 0.011 mbar for 24 h in a Christ BETA 2–8 LD plus freeze dryer (Osterode, Germany) until a constant weight was reached (5.79 g).

### 2.3. Preparation of Regenerated Biocomposite Films

Five different neat and biocomposite films with the following chemical compositions (Table 1) were prepared.

Cellulose (1000 mg) was dissolved in 20 mL [BMIM][Cl] at 110 °C under constant stirring for 2 h.

The PCL sample was similarly prepared by the dissolution of 1000 mg of ground PCL pellets in 20 mL [BMIM][Cl], heated up to 90 °C, and mechanically stirred at that temperature for 24 h.

To prepare CELL/PCL and CELL/PCL/KER blends, PCL or PCL and KER were added into the previously prepared CELL solution, and the respective mixtures were mechanically stirred for 24 h at 90 °C, as described in the literature [10].

The inclusion of GCC to form the CELL/PCL/KER/GCC blend was performed in two steps. The first step included ultrasonic dispersion of GCC in [BMIM][Cl] for 7 min using an ultrasonic homogenizer (VCX 750, SONICS, Newtown, NSW, Australia) with a 19 mm diameter probe tip at 20 kHz and 750 W output power to deagglomerate the as-delivered dewatered crumble-like press cake. After that, CELL was added to the GCC dispersion, heated up to 110 °C, and mechanically stirred at that temperature for 2 h. Thereafter, PCL and KER were added, and the mixture was mechanically stirred for 24 h at 90 °C.

The resulting homogenous blends were cast into thin layer films using a glass Petri dish. After casting, the samples were then immersed in deionized water to remove the water-miscible [BMIM][Cl] by extraction. After several washing cycles following immersion in water for a total of 24 h, the biocomposite films were air dried. Before testing, they were additionally dried for a further 24 h in a vacuum chamber.

The [BMIM][Cl] was finally recovered, firstly by the evaporation of water in a rotary evaporator (Heidolph Instruments, Schwabach, Germany) at 80 °C and 80 mbar for 4 h, each from 600 mL batches, until the majority of the water had been removed from both the immersion and washing solution as collected from the biocomposite gel regeneration stage, and secondly, after storing overnight at −20 °C, by freeze-drying at −60 °C under 0.011 mbar for 24 h until constant weight was reached (19.7 g from the 600 mL batch). The concentration of cellulose biopolymer in the ionic liquid (22.0 g) was fixed at 4.35% (*w*/*w*) (Figure S1, Supplementary Materials). The recycled [BMIM][Cl] could, thus, be used again as a solvent for cellulose dissolution.

### 2.4. Methods Used for Characterization

The morphology of the samples was studied using a Field Emission Scanning Electron Microscope operating at 20 kV, together with a microanalysis Tescan Mira3 XMU system (Tescan Orsay Holding AS, Brno, Czech Republic). Polymer configuration and spherulite properties were visualized using a Biological LED Polarization Optical Microscope (POM) (CX43, Olympus, Tokyo, Japan). Thermal infrared images were taken with a Fluke PTI120-9HZ Pocket Thermal Imager, and data handling was performed by the in-built thermal imaging software Fluke SmartView 4.3 (Fluke Corporation, Everett, WA, USA).

Attenuated Total Reflection Fourier-Transform Infrared (ATR–FTIR) spectrometry, Nicolet iS10 (Thermo Fisher Scientific Inc., Waltham, MA, USA), was used to detect the surface chemistry of the prepared biocomposite materials. Absorbance spectra were measured in the range 4000–500 cm^−1^. The spectral resolution was 4 cm^−1^ following 32 scans, and the results obtained from extracted keratin were analyzed using the spectral analysis software OMNIC^TM^ (Thermo Fisher Scientific Inc., Waltham, MA, USA) in the range 1600 cm^−1^–1700 cm^−1^.

Thermal behavior of the biocomposite films was examined using a differential scanning calorimeter (DSC-60Plus, Shimadzu, Kyoto, Japan). The temperature was increased from room temperature to 150 °C at a heating rate of 10 °C min^−1^. Using a starting sample weight of 5 ± 0.5 mg, the heat flow was monitored during heating under a nitrogen purge gas flow of 50 mL min^−1^.

Elemental analysis of powdered keratin was performed using an elemental analyzer (Vario EL III cube, Elementar Analysensysteme GmbH, Langenselbold, Germany), and the measured contents of N and S were found to amount to 12.78% and 3.72%, respectively [13].

The thickness of three specimens for each formulation (CELL, CELL/PCL, CELL/PCL/KER and CELL/PCL/KER/GCC) was measured at five points using an electronic caliper (pro-max Fowler, Fowler High Precision, Canton, MA, USA).

The light transmittance characteristic of blend films was investigated in the wavelength range of 200–800 nm using an ultraviolet–visible spectrophotometer (UV-2600, Shimadzu, Kyoto, Japan).

Film tensile behavior was analyzed using a texture analyzer (EZ Test LX Texture Analyzer, Shimadzu, Kyoto, Japan) operating in tensile mode with a 500 N load cell and a gauge length of 20 mm. The specimens (35 mm × 10 mm) were stretched in triplicate at a crosshead speed of 10 mm min^−1^. The tests were performed under 55% relative air humidity at 23 °C. The resulting stress–strain curves were used to determine tensile strength, elongation at break, and modulus of elasticity using the software Trapezium X (Shimadzu, Kyoto, Japan).

**Figure 2:**
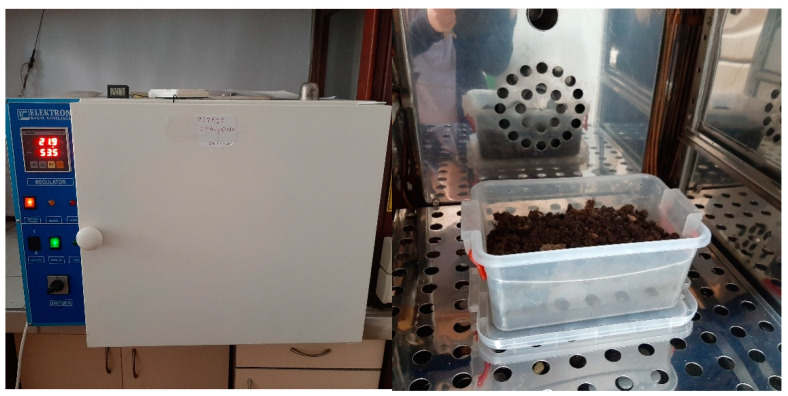
Soil degradation experiment set-up.

The biodegradability of the mulch films was tested in an in-soil degradation experiment carried out in an open polyethylene container (22 × 15 × 10 mm^3^) kept in a climate chamber (VIMS Elektron, Banja Koviljača, Serbia) conditioned at 23 °C and 50–55% relative humidity for 28 days (Figure 2). The soil used for this biodegradation experiment was produced by Solmax Kft (Külterület, Hungary), while packed in Domel d.o.o. (Belgrade, Serbia), with the commercial name Gardener. It is a universal soil for planting that contains nutrients in the amount of 50–300 mg L^−1^ nitrogen (N), 60–250 mg L^−1^ phosphorus (P_2_O_5_), and 80–400 mg L^−1^ potassium (K_2_O). Cellulose and biocomposite films were cut into a size of 20 × 10 mm^2^. The weight of each sample was recorded before burial (labeled as *W*_1_). After that, the films were buried in the soil at a depth of 25 mm from the surface.

Samples were removed from the soil at specific times (0, 7, 14, 21, and 28 days), washed with deionized water, and dried to a constant weight (*W*_2_). Weight loss (*W*_L_) was measured and taken as a percent of the biodegradability of each corresponding sample. The weight loss was calculated using the following Equation:

%*W*_L_ = [(*W*_1_ − *W*_2_)/*W*_1_] × 100 (1)

The content of calcium in CELL/PCL/KER/GCC composite films before and after the biodegradability test was determined with Inductively Coupled Plasma Optical Emission Spectrometry (ICP-OES). ICP-OES measurement was performed using a Thermo Scientific iCAP 6500 Duo ICP (Thermo Fisher Scientific, Cambridge, UK) spectrometer equipped with RACID86 charge injector device (CID) detector, standard glass concentric nebulizer, quartz torch, and alumina injector. Before ICP measurements, microwave digestion (total mineralization) of samples was performed in a microwave digester, Advanced Microwave Digestion System (ETHOS 1, Milestone, Sorisole, Italy), using an HPR-1000/10S segmented rotor operating under high pressure.

## 3. Results and Discussion

### 3.1. Cellulose Dissolution in [BMIM][Cl]

**Figure 3:**
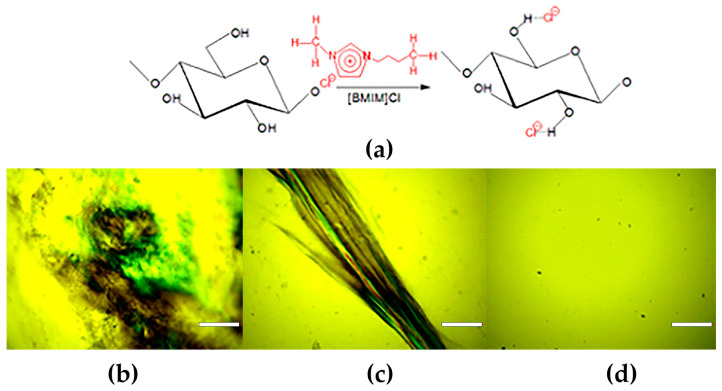
(**a**) Interaction between CELL and [BMIM][Cl] and optical micrographs of CELL dissolution in [BMIM][Cl] at T = 110 °C for (**b**) 30 min, (**c**) 60 min, and (**d**) 120 min (scale bar = 200 µm).

Considering that ionic liquids represent the most promising type of cellulose solvent, [BMIM][Cl], characterized by its superior dissolving capacity towards various polymers, was selected as a solvent for CELL and other film constituents such as PCL and KER. During the dissolution of CELL in [BMIM][Cl], the ionic liquid’s chloride anion (Cl^−^) interacts through hydrogen bonding with the hydroxyl groups present in the cellulose (Figure 3a), thus breaking the strong intermolecular hydrogen bonds between the polysaccharide chains, whereas the imidazolium cations of the ionic liquid play a relatively less important role [14].

Near the melting point of [BMIM][Cl] (about 65 °C), the viscosity of the solution is too high to enable satisfactory mixing, and the dissolution of cellulose takes a long time. Operating at 110 °C provided a suitable balance between [BMIM][Cl] viscosity reduction and thermal stability with ease in achieving complete cellulose dissolution. To establish the conditions for the efficient dissolution of cellulose in ionic liquid, the process was monitored using a polarization optical microscope. As is evident from Figure 3, a high activity of Cl^−^ present in the [BMIM][Cl] resulted in the dissolution of cellulose within 120 min of intensive mixing at 110 °C. On the other hand, during cellulose regeneration, water used as an anti-solvent preferentially forms hydrogen bonds with the Cl^−^ anion; consequently, the hydrogen bonds between cellulose units are re-established, leading to gel formation [15].

**Figure 4:**
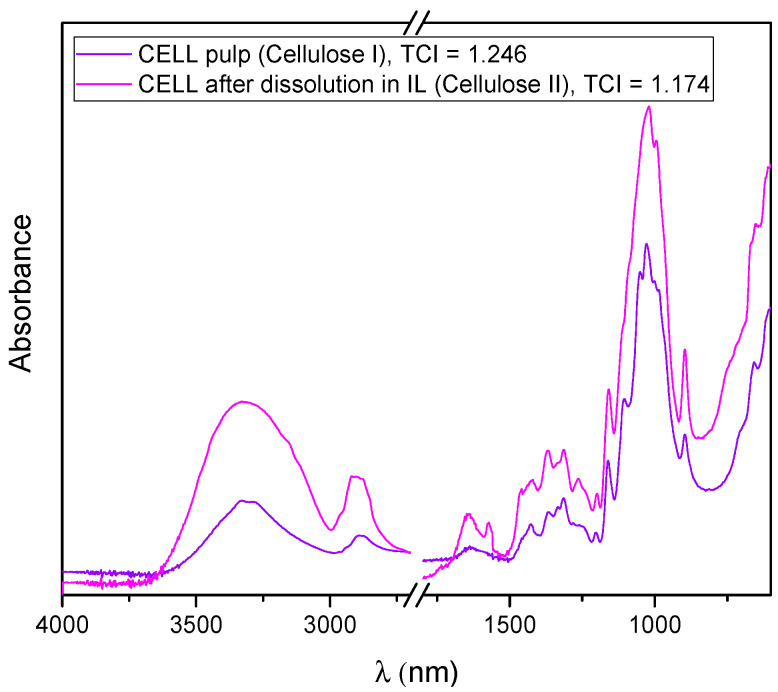
Comparison of ATR-FTIR spectra and total crystallinity index (TCI) of cellulose before and after dissolution and regeneration from [BMIM][Cl] solution.

Recorded FTIR spectra of cellulose before and after dissolution and regeneration from [BMIM][Cl] (Figure 4), i.e., the shifting and broadening of some bands, as well as a decrease in the total crystallinity index (1.246 vs. 1.174), highlighted that cellulose I was transformed to the more amorphous cellulose II. The TCI for cellulose I and cellulose II was calculated from the ratio of the intensities of the bands at 1372 and 2900 cm^−1^, and 1364 and 2892 cm^−1^, respectively [16].

### 3.2. Keratin Extraction from Chicken Feather Waste

**Figure 5:**
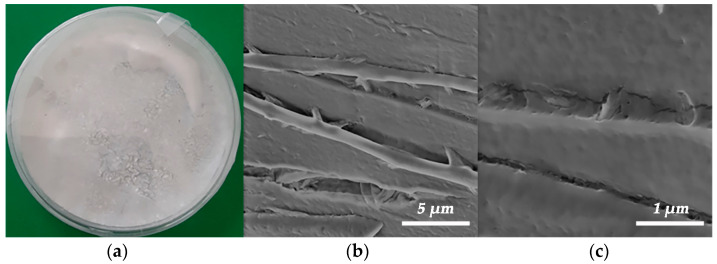
The appearance of the powdered keratin extracted from chicken feather waste (**a**) and its FE-SEM micrographs at different magnifications (**b**,**c**).

The visual appearance of the powdered keratin and its FE-SEM micrographs are presented in Figure 5. Keratin obtained by extraction from chicken feather waste according to the protocol given in Figure 1 can be considered as so-called hard keratin, since the content of sulfur (determined by elemental analysis) is higher than 3%. Keratin consists of two types of crystal structures, α-helix and β-sheet. On the one hand, the α-helix structure contributes to the elastic and flexure properties, whilst, on the other hand, the β-sheet structure is a crystal structure with a pleated-sheet form, providing rigidity. Such hard keratin is suitable for using as reinforcement and, hence, tailoring the mechanical properties of biocomposites [13,17]. The FE-SEM images reveal that the keratin protein shows a fibrous structure (Figure 5b,c) and consists of fibers with an average diameter of 1.25 ± 0.08 µm. The analysis of the average fiber diameters of keratin was performed using Image-Pro Plus 6.0 software (Media Cybernetics, Rockville, MD, USA).

The secondary structure of extracted keratin was identified using ATR-FTIR spectroscopy [18]. First, the spectrum was processed using the Fourier self-deconvolution of the OMNIC Software, and a baseline correction was performed. The amide I band in the ATR-FTIR spectrum of keratin (typically present within the range of 1700–1600 cm^−1^) was then predicted and deconvoluted with a Lorentzian line shape function, and the deconvoluted spectrum was fitted with individual Gaussian bands using Origin Pro 8.5 software (OriginLab, Northampton, MA, USA).

**Figure 6:**
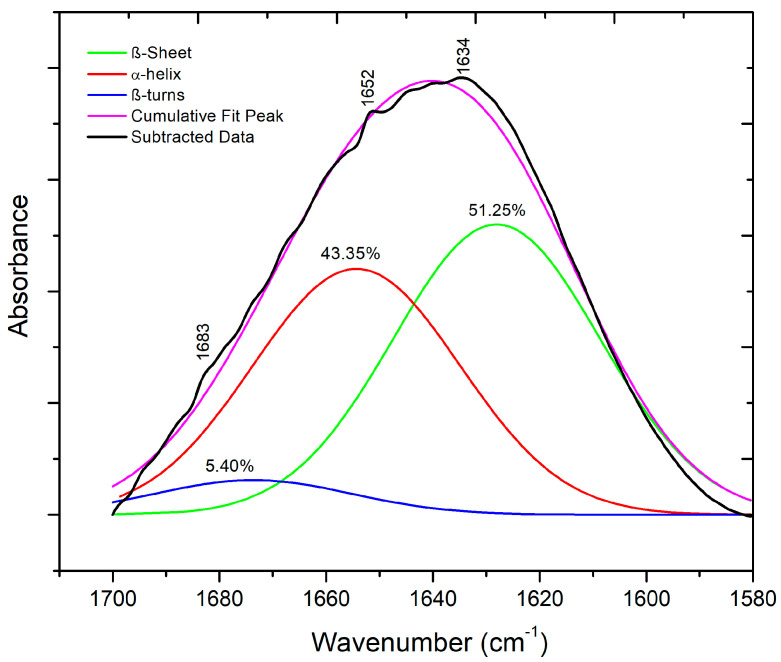
Deconvoluted amide I band of ATR-FTIR spectrum of extracted keratin.

Furthermore, based on the performed deconvolution of its ATR-FTIR spectrum (Figure 6), it can be stated that the secondary structure of extracted keratin mainly consists of β-sheet+ random coil conformation (51.25%) and α-helix (43.35%), with a lower content of β-turns (5.40%) [19].

### 3.3. Appearance of the Prepared Biocomposite Films and Their Optical Properties

**Figure 7:**
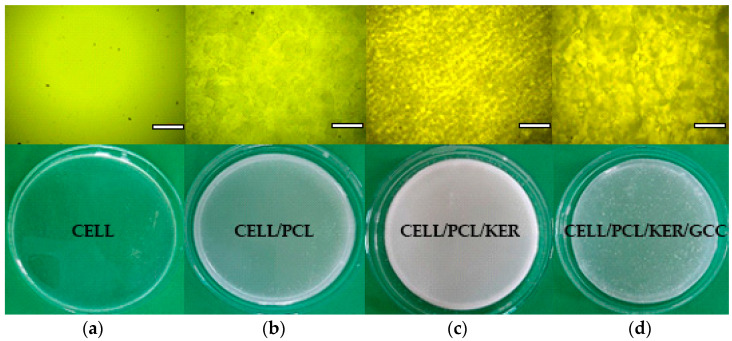
Optical micrographs and photographs of biocomposite films (**a**) CELL, (**b**) CELL/PCL, (**c**) CELL/PCL/KER, and (**d**) CELL/PCL/KER/GCC cast from their [BMIM][Cl] solutions (scale bar = 200 µm).

The preparation of CELL, CELL/PCL, CELL/PCL/KER, and CELL/PCL/KER/GCC biocomposite films by dissolution in ionic liquid and subsequent solution casting was followed by polarized light microscopy, Figure 7. The utilization of ultrasonication effectively reduced the initial agglomerates of GCC, Figure 7d. Furthermore, the ultrasonic process increased the dispersion temperature from 36.5 °C to 112.1 °C after 7 min, which was imaged using a thermal imaging camera (Supplementary Materials Figure S2). The SEM images presented in Figure S3 (Supplementary Materials) clearly show the difference in the appearance of the surface of different biocomposite films. After the regeneration of cellulose from ionic liquid, the surface morphology of the cellulose films was relatively smooth, and a homogeneous microstructure was observed. Microphase separation is obviously the greatest for the CELL/PCL blend films. The CELL/PCL/KER biocomposite films are relatively smooth, probably due to better microphase miscibility, while visual inspection confirmed surface roughness after the addition of CaCO_3_ filler (Figure 7d).

**Figure 8:**
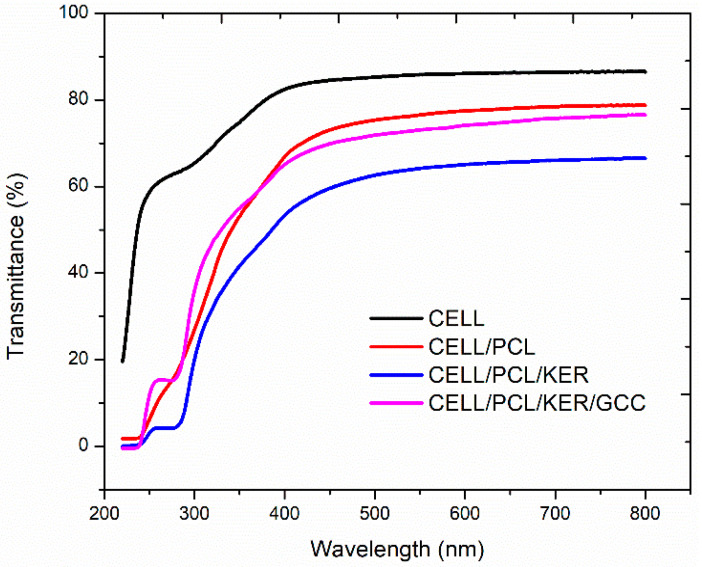
Transmittance spectra of examined neat cellulose and biocomposite films.

The regenerated cellulose film (CELL) exhibited a highlight transmittance (about 86.0% at 600 nm) in the visible region, which is in line with the results published by Zheng et al. [15]. With the addition of PCL and especially PCL and KER to the CELL, the transmittance of the prepared films decreased down to 77.9% and 65.3% at 600 nm, respectively. However, the incorporation of GCC filler, as can be seen from Figure 7d and Figure 8, contributed to an increased transmittance (74.4% at 600 nm), very similar to that of CELL/PCL. The visible light transmittance across the broader spectrum, Figure 8, is only marginally altered for CELL/PCL/KER/GCC with GCC versus the CELL/PCL/KER without GCC. Figure 8 shows a notable decrease in the light transmittance at longer wavelengths from 86.0% to 65.3% for biocomposite films with 20% (*w*/*w*) of keratin, which suggests that by adding a higher content of keratin, we can make films with low transparency. Such mulch films can also reduce weed growth due to their antimicrobial properties [20] and prevent the penetration of photosynthetically active radiation (PAR), which is needed for the weed seedlings to grow. Transparent mulch films, such as CEL/PCL/KER/GCC, can alternatively be used for partial soil sterilization by covering the soil in the absence of crops for several weeks during the hottest period of the year [21].

### 3.4. ATR-FTIR Spectroscopy of Biocomposite Films

**Figure 9:**
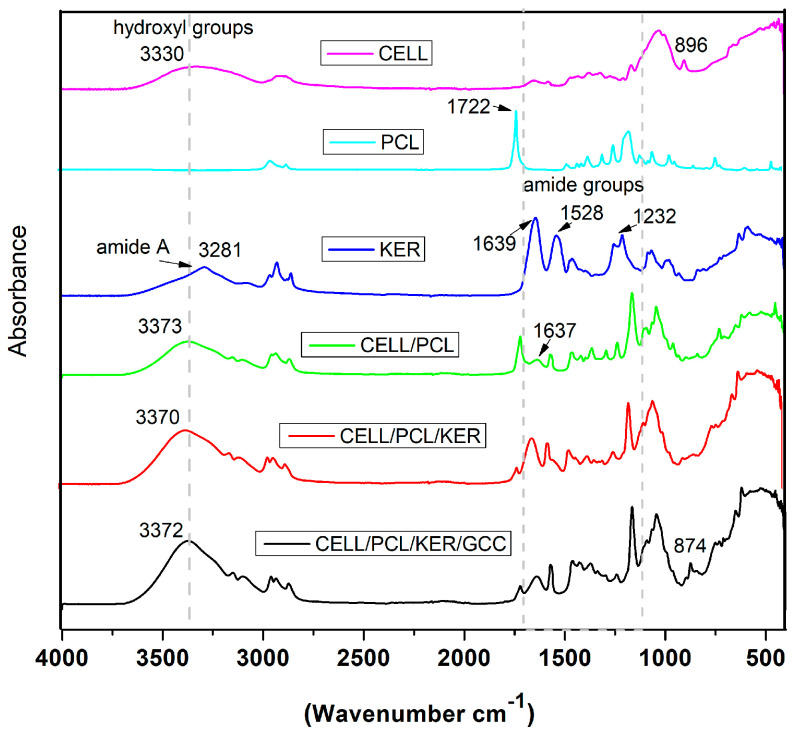
FTIR spectra of CELL, PCL, KER, and biocomposite films cast from their [BMIM][Cl] solutions.

ATR-FTIR spectroscopy was used to reveal interactions between components in the investigated biocomposite films. FTIR spectra (Figure 9, Table S3 (Supplementary Materials)) show the existence of inter- and intramolecular hydrogen bonds between cellulose and the blend components. The samples were scanned at 4000–400 cm^−1^, but the regions of interest are the OH stretch region in the 3000–3700 cm^−1^ range and the amide region at 1100–1710 cm^−1^. After the [BMIM][Cl] dissolution of cellulose, the destruction of inter- and intramolecular hydrogen bonding was observed in the amorphous region, and the existence of glycosidic linkage at 896 cm^−1^ cm was detected [22].

The KER sample showed spectral bands corresponding to peptide bonds (-CONH) that were identified as amide A and amide I–III. The peptide bond amide A vibrations exhibited an absorption band at 3281 cm^−1^, assigned to the N–H stretching vibration. The bands at 1639 cm^−1^, 1528 cm^−1^, and 1232 cm^−1^ were attributed to the C=O stretching vibration (amide I), C–N stretching and N–H bending vibrations (amide II), and C–N and C–O stretching and N–H and O=C–N bending vibrations (amide III), respectively [13]. In the area of amide groups, the major absorption bands of the components overlap in the case of these biopolymers. The spectrum of the PCL sample showed the characteristic peaks of PCL attributed to crystalline carbonyl stretching (−C=O) located at 1722 cm^−1^ and shifted to the lower wavenumber region for CELL/PCL films. In the case of partially miscible biopolymers, such as CELL/PCL in ionic liquid, the new peak at 1637 cm^−1^ can be assigned to the carbonyl groups of PCL interacting with the hydroxyl groups of cellulose through hydrogen bonding [10]. In samples containing keratin (CELL/PCL/KER and CELL/PCL/KER/GCC), the increase in the absorption peak, compared to pure regenerated cellulose, in the hydroxyl and amide A region is likely due to the bonding between the -NH groups of keratin and the -OH groups of cellulose, which displays an increase in the intermolecular hydrogen bonds [23], as presented in the FTIR spectra, Figure 9. The FTIR spectra for semi-crystalline polymers, such as cellulose, can be split into two peaks, corresponding to the amorphous and crystalline phases. The increase in the cellulose II (regenerated cellulose), with total crystallinity index (TCI, Table 2), i.e., ratio of the intensity of bands at 1372 cm^−1^ and 2900 cm^−1^, was directly proportional to the crystallinity degree of the samples [24], rising from 1.17 for CELL to 2.13 for CELL/PCL/KER/GCC, confirming that the addition of PLC, PLC/KER, and PCL/KER/GCC positively influences cellulose crystallinity. In addition, these additions increased both the lateral order index (LOI), i.e., the absorbance ratio of the bands at 1418 cm^−1^ and 896 cm^−1^ [24,25], and the hydrogen bond intensity (HBI), a ratio of the peaks at 3336 and 1336 cm^−1^ of cellulose II related to the level of hydrogen bonding [24].

### 3.5. Thermal and Mechanical Properties of the Prepared Biopolymers and Biocomposite Films

**Figure 10:**
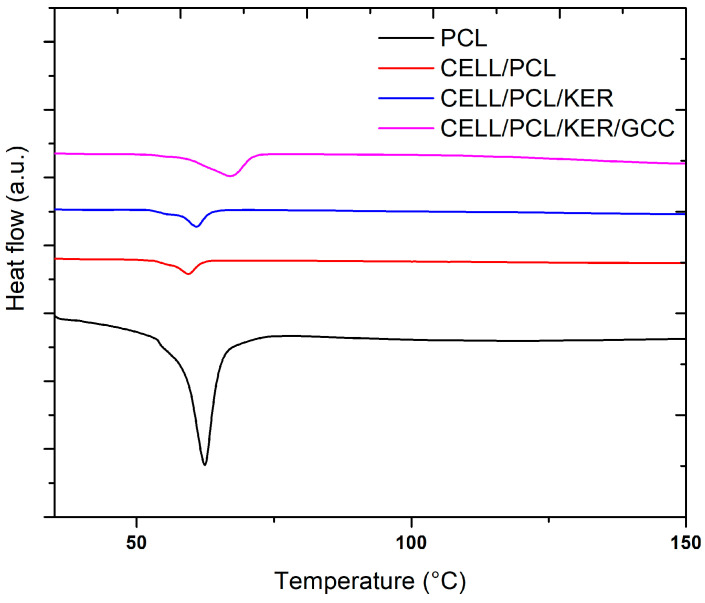
DSC analysis of the neat PCL and biocomposite films.

The miscibility of the three examined biopolymers (CELL, PCL, and KER) has a great effect on the thermo-mechanical properties of the biocomposites, which are predominantly controlled by inter- and intramolecular interactions. The main thermal parameters, including the melting temperature (*T_m_*), the melting enthalpy (∆*H_m_*), and the degree of crystallinity (*X_m_*), for the investigated biocomposite films are shown in Table 3. The crystalline properties of the CELL/PCL films decreased owing to the molecular chains of cellulose sterically inhibiting the crystallization of PCL. Moreover, melting point temperature depression indicates the lower crystallinity of CELL/PCL and the formation of less perfect crystals, both of which are characteristic for partially miscible polymer blends [10,26]. Performed DSC analysis showed that the addition of the two components KER/GCC to the PCL also led to a decrease in the crystallinity of the PCL component in these biocomposite films, Table 3 and Figure 10. Conversely, biocomposite films containing these same components, KER/GCC, show an increase in melting temperature (Table 3), which is possibly due to the combination of two phenomena: mutual macromolecular interaction of the starting components (Figure S4, Supplementary Materials) and increased heat capacity when GCC is present, resulting in heating delay due to thermal absorption, i.e., an effect of the amount of thermal energy required to raise the temperature and so dependent on the rate of heating (Figure S5, Supplementary Materials). A higher melting temperature of the combination CELL/PCL/KER versus both PCL alone and in the CELL/PCL blend is mostly influenced by the presence of cellulose, while the secondary structures within the keratin contribute to the amorphous regions [27].

The equation for the degree of crystallinity (*X*_m_) calculation is as follows:

(2) $$\chi_{m,\mathrm{sample}\left(\%\right)}=\frac{\Delta H_{m,\mathrm{sample}}}{\Delta H{{}^{\circ}}_{m,\mathrm{PCL}}}\times 100$$

where Δ*H*_m,sample_ is the melting enthalpy of the various samples ranging across neat PCL and its biocomposites, while Δ*H*°_m,PCL_ is the literature value of melting enthalpy of 100% crystalline PCL (139.5 J g^−1^) for the PCL molecular weight of 80,000 g mol^−1^, according to Radisavljevic et al. [28].

**Figure 11:**
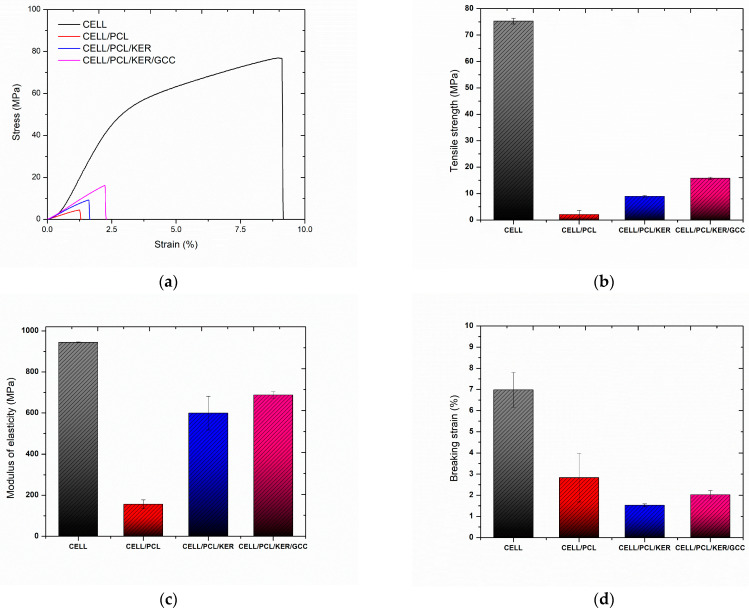
Mechanical properties of cellulose and biocomposite films. (**a**) Stress against strain curves, (**b**) tensile strength (MPa), (**c**) modulus of elasticity (MPa), and (**d**) breaking strain (%).

The following presented results revealed that the studied biocomposite films have different fine structures, which, together with the different miscibility of the three biopolymers and their inter- and intramolecular interactions, represent essential factors affecting the film mechanical properties, such as tensile strength, modulus of elasticity, and breaking strain, which are presented in Table 4 and Figure 11. The mechanical properties of the cellulose films decreased with the addition of PCL polymer, which caused fragility of the blend films. The biocomposite films prepared with the addition of a small content of keratin form strong intermolecular bonding interactions between the components of the films. Taking advantage of the good solubility of both cellulose and keratin in ionic liquids, many authors studied the mechanical properties of these systems for polysaccharide/polypeptide film formation. Shamsuri et al. [26] and De Silva et al. [23,29] published a series of studies on prepared regenerated cellulose/feather composite films and fibers, which also showed enhanced mechanical properties with an optimum keratin amount of 10% (*w*/*w*). However, in this case, the authors attribute this effect to the amount of α-helix keratin improving the elastic properties in the composite films. Kammiovirta et al. [5] additionally observed that whilst 10% (*w*/*w*) of keratin addition into the cellulosic filaments improved the mechanical properties, higher keratin addition levels resulted in reduced mechanical performance. Tran et al. [30] also investigated composites with cellulose and wool keratin by dissolution in [BMIM][Cl] ionic liquid. They showed that wool keratin has a larger number of α-helix structures than feather keratin. However, the α-helices were disrupted by dissolution in ionic liquid, while the amount of β-sheet conformation increased during the regeneration process of cellulose and keratin. They observed that β-sheet conformation affects the increase in tensile strength (~38 MPa) in the cellulose/keratin composites by 40% (*w*/*w*) keratin loading [30]. Hydrophobic and hydrophilic surfaces of extracted keratin can act as a “bridge” for the blending and bonding between the incompatible hydrophilic cellulose and hydrophobic PCL, which can be assumed to be because there is an increase in the modulus of elasticity and tensile stress comparing CELL/PCL/KER containing keratin to CELL/PCL samples without keratin [31].

The addition of PCL and PCL/KER to CELL decreased the tensile strength and modulus of elasticity of the resulting biocomposite films, while the addition of 10% (*w*/*w*) GCC significantly increased the composite CELL/PCL/KER/GCC tensile strength and modulus (Table 4). This improvement in mechanical properties may be explained by the ultrasonic surface modification of GCC by [BMIM][Cl] and the fact that these modified particles with a lower particle size act as physical cross-linkers between regenerated cellulose molecules [32], promoting the crystallization and orientation of the cellulose polymer (Table 2).

### 3.6. Biodegradability of Developed Cellulose and Biocomposite Films

**Figure 12:**
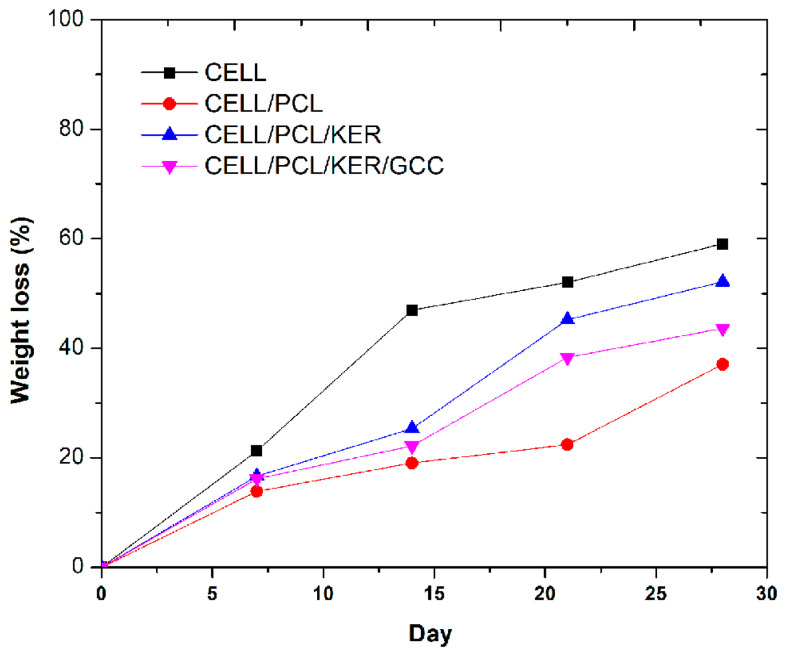
Weight loss, *W*_L_, of cellulose and biocomposite films during a 4-week period of soil-burial.

The biodegradation properties of cellulose and biocomposite films were investigated by a soil degradation experiment. The weight loss curves and the pictures of the films’ surfaces during the biodegradability test are included in Figure 12 and Table 5, respectively. According to the image evolution from Table 5, all PCL-based biocomposite films retained their shape for 2 weeks, except for the cellulose film. The films containing KER and GCC showed color changes from white to yellow 21 days after the start of the biodegradability test, and obvious holes and cracks appeared on the surface of the biocomposite, while the cellulose film degraded faster. After 4 weeks of burial, the holes on the entire surface of the biocomposite were further expanded, while the cellulose and CELL/PCL film began to disintegrate.

The biodegradability of the cellulose and biocomposite films was determined by weight loss during soil burial tests according to Al Hosni et al. [11]. Cellulose has a higher crystallinity in its original state prior to dissolution, while the crystallinity of the regenerated cellulose was seen to decrease upon ionic liquid dissolution, according to Figure 2 and Figure 3. Moreover, the specific surface area and the number of accessible hydrophilic groups increased after the action of dissolution, resulting in the fast degradation of regenerated cellulose film when compared with other biocomposites. The weight loss, *W*_L_, of cellulose film was 59% after 4 weeks of being buried in soil, indicating that regenerated cellulose film has excellent biodegradability. By the addition of PCL polymer, the degradation rate (weight loss, *W*_L_) of CELL/PCL samples is reduced to 37% after 4 weeks of being buried in soil, which can be explained by the lower biodegradability of PCL. Guarás et al. [33] reported that PCL buried in soil reached only 1% *W*_L_ after 21 days and achieved only 3% *W*_L_ after 243 days of the assay. However, PCL showed a fast degradation rate when buried in compost and incubated at 50 °C; it reportedly degrades completely with microorganisms within 91 days [11].

The addition of KER to CELL/PCL increased the degradation rate of these multicomponent biocomposite films. In the first week, about 17% and 16% by weight of CELL/PCL/KER film and CELL/PCL/KER/GCC film was lost, respectively; in the following 2 weeks, CELL/PCL/KER lost 25% and 45% of its weight each week, and the CELL/PCL/KER/GCC film lost 22% and 38% of its weight each week. After 4 weeks of simulated degradation in soil, the mass residual rates of the CELL/PCL/KER film and CELL/PCL/KER/GCC film were 52% and 44%, respectively (Table S1, Supplementary data). These obtained results can be explained by the fact that keratin possesses a large number of hydrophilic hydroxyls, carboxyl, and amino groups that enhance the biodegradability of the CELL/PCL/KER film, with, in this case, a keratin content of 20% (*w*/*w*). All samples with keratin that were buried in the soil for four weeks showed microorganism growth and intensive biodegradation (Table 5, images c and d). Soil is considered a well-known source for the growth of keratinophilic microflora (bacteria and fungi), which can degrade keratin waste with the production of the microbial keratinase enzyme. Filippello-Marchisio [34] pointed out that the role of keratinophilic fungi in the degradation of keratin substrates is mainly due to the presence of proteolytic enzymes called keratinases, which are present in the natural soil environment. Li et al. [35] showed that keratin-sprayed mulch films show high biodegradability and so open a new direction for the manufacture of plant nutritional mulch films in ecological agriculture. The degraded keratin can serve as a source of nitrogen, sulfur, and carbon for soil microorganisms and can be used as a fertilizer for plant growth. However, to improve the mechanical properties to those required by agricultural mulch film, a biodegradable polymer or inorganic particles must still be used for the preparation of keratin-based biocomposites [36]. In the field of agriculture, GCC is also used as a fertilizer to supply calcium to plants and to stabilize the pH value, thereby reducing acidic conditions in the soil, in our case from pH 4–5 to pH 5–6 after the biodegradation of the samples. The biodegradation of the mulch was also studied by measuring Ca^2+^ ion release in parallel with the weight loss of the samples during 28 days of treatment in the soil (Table S2, Supplementary data). A continuous Ca^2+^ ion release is evident for CELL/PCL/KER/GCC mulch film related to the material biodegradation after 28 days.

It was found that the biocomposite film containing GCC showed a slight reduction in biodegradation rate in soil, probably due to the increased crystallinity of the films, compared to other biocomposite films (Table 3), confirming that the degree of crystallinity of a biopolymer(s) is one of the important factors that affects the biodegradation process.

## 4. Conclusions

Biodegradable mulch films from unique combinations of cellulose (CELL), cellulose blended with polycaprolactone (CELL/PCL), plus additional keratin (CELL/PCL/KER), and with the introduction of ground calcium carbonate (GCC) functional filler (CELL/PCL/KER/GCC) were prepared using ionic liquid (1-butyl-3-methylimidazolium chloride [BMIM][Cl]) as a green solvent. Miscibility; morphological, physicochemical, thermal, and mechanical properties; and biodegradability were evaluated in terms of the respective film composition. The structural properties of the α-helix and β-sheets of the extracted KER were investigated using a Fourier transform spectroscope in an IR absorption mode, applying deconvolution methods to determine the secondary structure of the polypeptide. The mechanical strength and elastic properties of the resulting biocomposites were less than those of the pure cellulose biopolymer alone due to the structural segregation arising from the limited partial miscibility within the polymer blends. The opposite effect was observed upon the addition of KER and GCC, with an increased modulus of elasticity and tensile stress response compared to the dual-polymer CELL/PCL blends. The light transmittance of the mulch film at different ratios of keratin addition was noticeably lower than that of the single CELL and CELL/PCL films. The biodegradation test also indicated that the incorporation of KER enhanced the biodegradability of the biocomposites, while GCC addition slightly inhibited degradation, partly due to an increased hydrophobicity and crystallization growth of semi-crystalline biopolymers. This work illustrates a successful design path, including the use of environmentally friendly recoverable ionic liquid for cellulose dissolution and regeneration, toward obtaining functional biodegradable mulch films that can be integrated into the soil during application and contribute to its enrichment with organic/inorganic fertilizing activity after biodegradation. The incorporation of natural cellulose biomass and keratin from chicken feather waste in the formulation has several advantages, such as a reduction in costs and the valorization of agricultural waste, which promotes a healthy environment and a circular bioeconomy. For this reason, in the future, continuing research on mulch films, different polysaccharides, and polypeptides will be applied for the preparation of agricultural mulch entirely on a biological basis, without the use of synthetic biopolymers.

## Figures and Tables

**Table 1:** Abbreviations for films and their chemical compositions.

Film Abbreviation	CELL/PCL/KER/GCC Amount (mg)
CELL	1000/0/0/0
PCL	0/1000/0/0
CELL/PCL	600/400/0/0
CELL/PCL/KER	400/400/200/0
CELL/PCL/KER/GCC	400/400/100/100

**Table 2:** Cellulose II total crystallinity index (TCI), lateral order index (LOI), and hydrogen bond intensity (HBI) calculated based on the absorbance FTIR spectra.

Sample	TCI	LOI	HBI
CELL	1.17	0.52	1.38
CELL/PCL	1.92	1.07	1.83
CELL/PCL/KER	1.52	0.93	1.99
CELL/PCL/KER/GCC	2.13	1.43	1.70

**Table 3:** Thermal properties of neat PCL and biocomposite films.

Sample	Melting Temperature (°C)	Melting Enthalpy (J g^−1^)	Degree of Crystallinity (%)
PCL	62.3	66.3	47.5
CELL/PCL	61.0	9.80	7.03
CELL/PCL/KER	62.6	21.9	15.7
CELL/PCL/KER/GCC	68.9	30.2	21.6

**Table 4:** Mechanical properties and thickness of neat cellulose and biocomposite films.

Sample	Tensile Strength (MPa)	Modulus of Elasticity (MPa)	Breaking Strain (%)	Thickness (mm)
CELL	75.3 ± 1.1	944.4 ± 2.0	6.97 ± 0.82	0.19 ± 0.04
CELL/PCL	2.1 ± 1.6	156.0 ± 21.5	2.83 ± 1.15	0.39 ± 0.03
CELL/PCL/KER	9.3 ± 0.3	599.1 ± 81.9	1.52 ± 0.07	0.37 ± 0.02
CELL/PCL/KER/GCC	15.8 ± 0.4	687.5 ± 16.6	2.03 ± 0.20	0.40 ± 0.06

The tensile properties of PCL neat film are not able to be studied because of its highly brittle behavior. All the samples were analyzed in triplicate, and the results are expressed as average ± standard deviation (SD).

**Table 5:** Biodegradability assay images of different samples, (**a**) CELL, (**b**) CELL/PCL, (**c**) CELL/PCL/KER, and (**d**) CELL/PCL/KER/GCC, removed at 0, 7, 14, 21, and 28 days (sample size 20 × 10 mm^2^).

	(a)	(b)	(c)	(d)
**0 week**	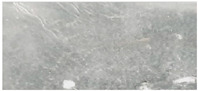	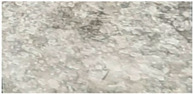	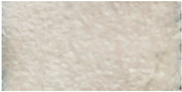	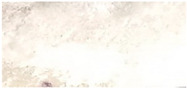
**1 week**	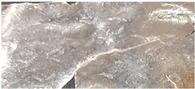	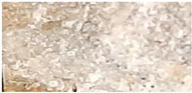	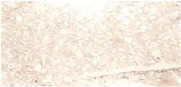	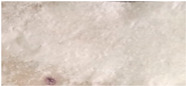
**2 week**	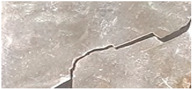	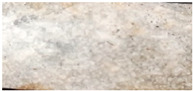	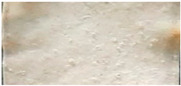	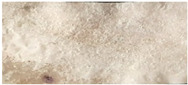
**3 week**	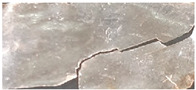	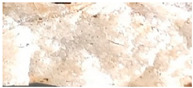	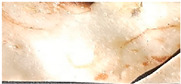	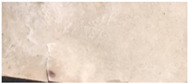
**4 week**	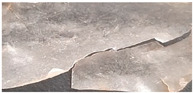	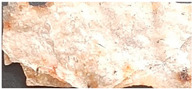	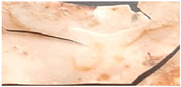	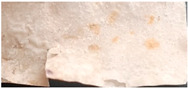

## Data Availability

The data presented in this study are available on request from the corresponding author.

## Supplementary Materials

The following supporting information can be downloaded at https://www.mdpi.com/article/10.3390/polym15122729/s1, Figure S1: Regeneration of ionic liquid [BMIM][Cl] (1-Butyl-3-methylimidazolium chloride): left, aqueous solution of [BMIM][Cl] obtained after mulch film regeneration and extraction/washing; right, regenerated [BMIM][Cl], m1-mass of [BMIM][Cl] used in experiment, and m^2^-mass of regenerated [BMIM][Cl]; Figure S2: Thermal infrared images of ultrasonic dispersion of the GCC in [BMIM][Cl] solution. The temperature of the dispersion increased from 36.5 °C to 112.1 °C after 7 min exposure to ultrasonication; Figure S3: FE-SEM micrographs of the surfaces of (a) CELL, (b) CELL/PCL, (c) CELL/PCL/KER, and (d) CELL/PCL/KER/GCC (scale bar = 200 µm); Figure S4: DSC analysis of (a) neat cellulose (CELL) and (b) keratin (KER); Figure S5: DSC analysis of CELL/PCL/KER/GCC biocomposite films at different heating rates: 5 and 10 °C min^−1^; Table S1: The weight loss (WL (%)) of the cellulose and biocomposite films during the biodegradability test; Table S2: The total content of calcium ions (Ca^2+^) in the mulch films (mg g^−1^ and % (*w*/*w*)); Table S3: ATR-FTIR absorption bands characteristic of biopolymers and biocomposites.
