# Supplementary material for: Biodegradable Cellulose/Polycaprolactone/Keratin/Calcium Carbonate Mulch Films Prepared in Imidazolium-Based Ionic Liquid
Source: Polymers (Basel). 2023 Jun 18;15(12):2729. doi: 10.3390/polym15122729 (PMC10301129; doi:10.3390/polym15122729)
Supplement: Supplementary file 1 [file polymers-15-02729-s001.zip › polymers-2454353-supplementary.pdf]

Biodegradable cellulose/polycaprolactone/keratin/calcium carbonate mulch films prepared in imidazolium-based ionic liquid

Dušica Stojanović[1], Aleksandra Ivanovska[2,*], Nemanja Barać[2], Katarina Dimić-Misić[3], Mirjana Kostić[1], Vesna Radojević[1], Djordje Janaćković[1,2], Petar Uskoković[1], Ernest Barceló[3] and Patrick Gane[1,3,*]

[1]Faculty of Technology and Metallurgy, University of Belgrade, Karnegijeva 4, 11000 Belgrade, Serbia; duca@tmf.bg.ac.rs (D.S.); kostic@tmf.bg.ac.rs (M.K.); vesnar@tmf.bg.ac.rs (V.R.); nht@tmf.bg.ac.rs (D.J.); puskokovic@tmf.bg.ac.rs (P.U.)

[2]Innovation Center of the Faculty of Technology and Metallurgy in Belgrade Ltd., University of Belgrade, Karnegijeva 4, 11000 Belgrade, Serbia; nbarac@tmf.bg.ac.rs

[3]Department of Bioproducts and Biosystems, School of Chemical Engineering, Aalto University, 00076 Helsinki, Finland; katarina.dimic-misic@metsagroup.com (K.D.-M.); ebarcelorodriguez@gestamp.com (E.B.)

* Correspondence: aivanovska@tmf.bg.ac.rs (A.I.); patrick.gane@aalto.fi (P.G.)

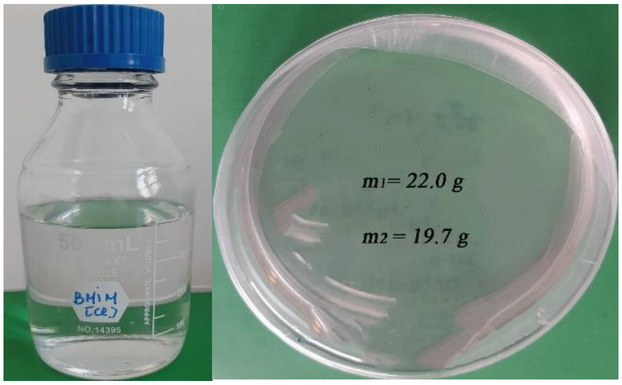

Figure S1. Regeneration of ionic liquid [BMIM][Cl] (1-Butyl-3-methylimidazolium chloride): left - aqueous solution of [BMIM][Cl] obtained after mulch film regeneration and extraction/washing; right - regenerated [BMIM][Cl], m_1-mass of [BMIM][Cl] used in experiment and m_2-mass of regenerated [BMIM][Cl]

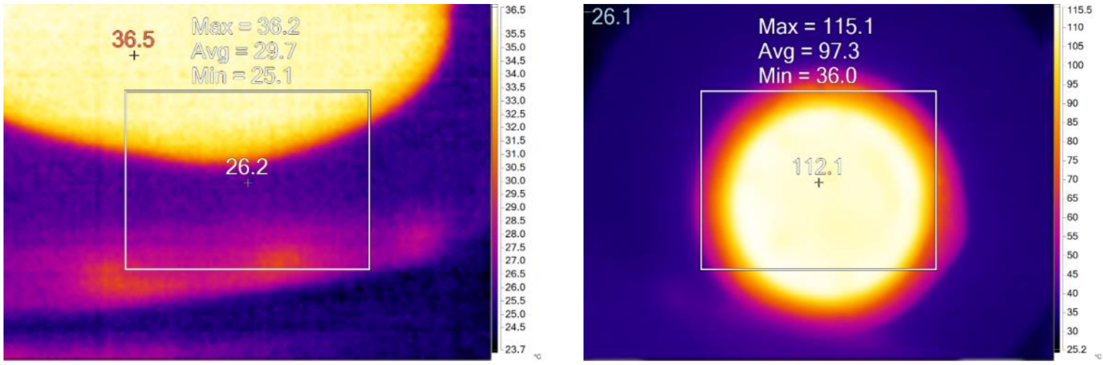

Figure S2. Thermal infrared images ultrasonic dispersion of the GCC in [BMIM][Cl] solution. The temperature of the dispersion increased from 36.5 °C to 112.1 °C after 7 min exposure to ultrasonication.

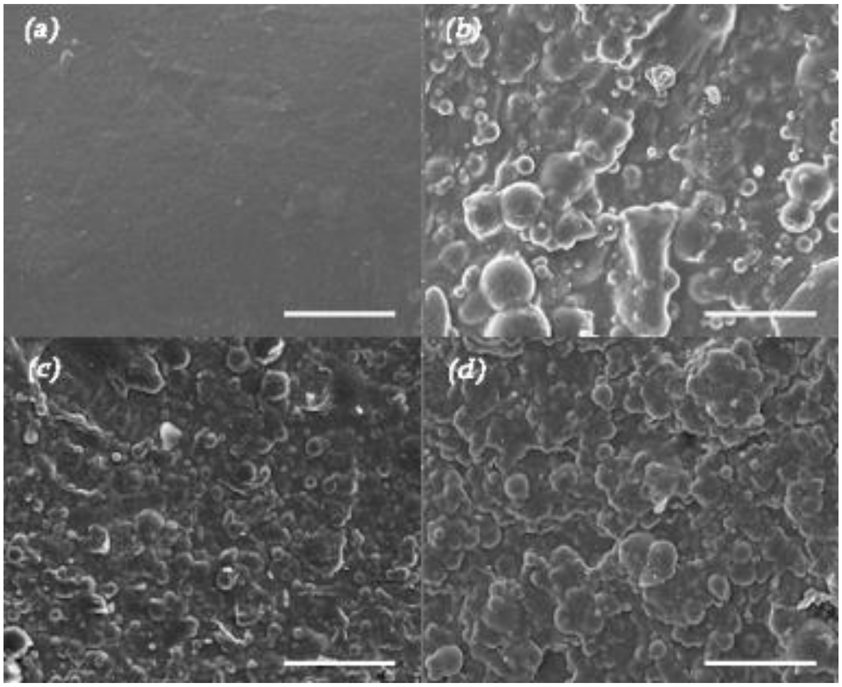

Figure S3. FE-SEM micrographs of the surfaces of (a) CELL, (b) CELL/PCL, (c) CELL/PCL/KER, and (d) CELL/PCL/KER/GCC (scale bar = 200 μm)

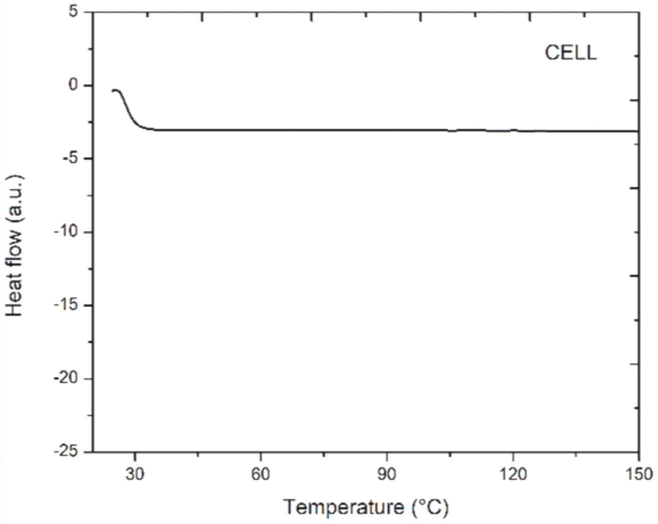

(a)

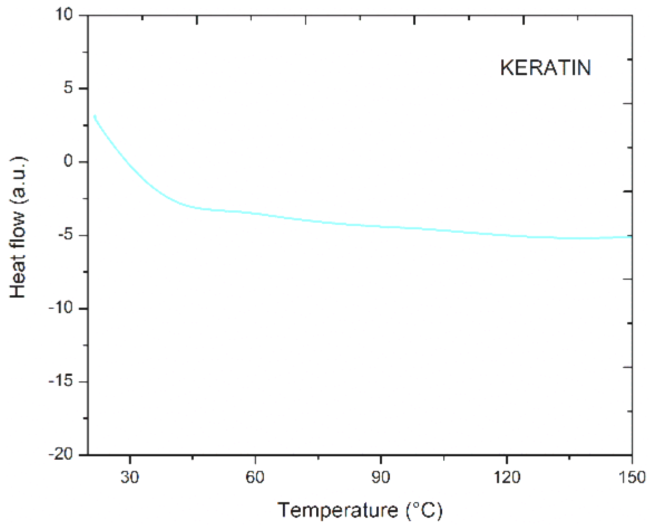

(b)

Figure S4. DSC analysis of (a) neat cellulose (CELL), and (b) keratin (KER).

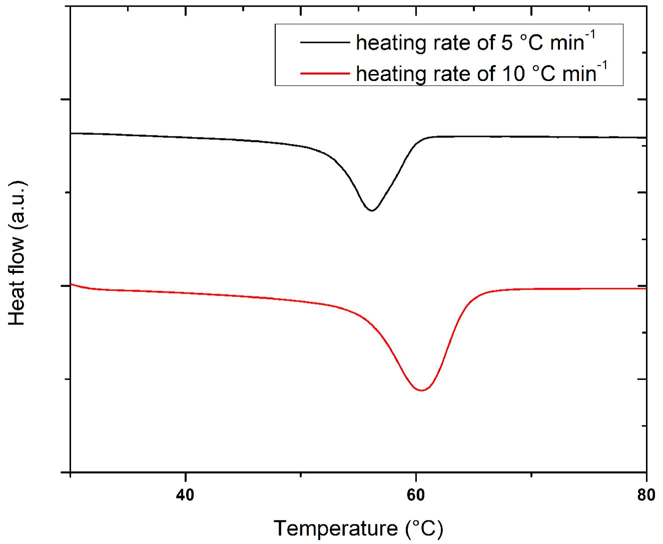

Figure S5. DSC analysis of CELL/PCL/KER/GCC biocomposite films at different heating rates: 5 and 10 °C min^{-1}

Table S1 The weight loss (W_L (%)) of the cellulose and biocomposite films during the biodegradability test

	W_L (%)			
**Day**	**CELL**	**CELL/PCL**	**CELL/PCL/KER**	**CELL/PCL/KER/GCC**
**7**	21.22	13.86	16.65	16.16
**14**	46.96	19.03	25.34	22.18
**21**	52.03	22.40	45.22	38.32
**28**	59.12	37.06	52.18	43.62

Table S2 The total content of calcium ions (Ca^{2+}) in the mulch films (mg g^{-1} and % (w/w))

**Sample**	**Total content of Ca^{2+} ions in the mulch films**	
	**mg g^{-1}**	**% (w/w)**
**CELL/PCL/KER/GCC (0 days)**	19.889	1.989
**CELL/PCL/KER/GCC (28 days)**	0.562	0.056

Table S3 ATR-FTIR absorption bands characteristic of biopolymers and biocomposites

**Wavenumber, cm^{-1}**	**Functional group**	**Components**
3000–3700	OH stretching	all
3281	N–H stretching	KER
1722	-C=O stretching	PCL
1100–1710	amide region	KER
1639	C=O stretching (amide I)	KER
1637	-C=O stretching	CELL/PCL
1528	C–N stretching and N–H bending (amide II),	KER
1232	C–O stretching and N–H and O=C–N bending (amide III)	KER
1418	Hydrogen intramolecular bonds	CEL
896	β-glycosidic linkage	CEL
874	C–H out of plane glucose ring	CEL
